# Early Hyperbaric Oxygen Therapy Promotes Recovery of Blunt Liver Injury in Rats via Inhibiting Inflammatory Response and Oxidative Stress

**DOI:** 10.7150/ijms.109842

**Published:** 2025-04-13

**Authors:** Houyu Zhao, Tingting Zhang, Yan Wang, Yiqun Fang

**Affiliations:** 1 Department of Diving Medicine, Navy Special Medical Center, Naval Medical University, Shanghai 200050, China.; 2 Department of Underwater and Hyperbaric Medicine, Navy Special Medical Center, Naval Medical University, Shanghai 200050, China.; 3 Department of Ultrasound, The 985th Hospital of the Joint Logistics Support Force of the Chinese People ' s Liberation Army, Taiyuan 030001, China.

**Keywords:** blunt liver injury, hyperbaric oxygen therapy, oxidative stress, inflammatory response

## Abstract

**Purpose:** Blunt Liver Injury (BLI) is a common form of blunt abdominal trauma, with most cases managed non-surgically in current clinical practice. However, the absence of targeted treatments addressing the pathological changes associated with liver injury can result in prolonged recovery and potential long-term complications. Hyperbaric oxygen therapy (HBOT), known for its anti-inflammatory and antioxidant effects, has shown therapeutic potential in various diseases. This prospective randomized controlled experimental animal trial aimed to evaluate the effect of HBOT on BLI in a rat model.

**Methods:** We established a BLI rat model by employing a free-fall weight method. Following injury, one group received no intervention, while the other was treated with HBOT (2.5 ATA, 60 minutes per session, once daily). Liver tissue and serum samples were collected at multiple time points for histological evaluation (HE staining), apoptosis detection (TUNEL staining), proliferation assessment (Ki67 immunohistochemical staining), and measurements of liver transaminase (ALT, AST), inflammatory markers (IL-1β, IL-6, TNF-α), and oxidative stress indicators (SOD, MDA, GSH).

**Results:** The results indicated that HBOT significantly reduced liver transaminase elevation, pathological damage, and hepatocyte apoptosis while promoting hepatocyte proliferation and accelerating liver function recovery. Mechanistically, HBOT alleviated oxidative stress and inflammatory response, highlighting its therapeutic potential.

**Conclusion:** These findings provide further evidence supporting the application of HBOT in the clinical management of BLI. This study helps advance the clinical approach to BLI by shifting the focus from symptom management to mechanism-based treatment.

## Introduction

Blunt liver injury (BLI) is a common type of abdominal trauma, frequently resulting from external forces such as traffic accidents and falls from heights^1^. In recent years, the incidence of BLI has increased, largely attributed to advancements in diagnostic technologies such as CT imaging. BLI induces multiple pathological changes in the liver^2^. The first is the inflammatory response, where blunt trauma rapidly triggers inflammation characterized by the recruitment of inflammatory cells and the release of pro-inflammatory cytokines^3^. Oxidative stress is another critical pathological feature following BLI. Under normal conditions, the liver possesses a robust antioxidant system, including superoxide dismutase (SOD) and glutathione peroxidase (GSH-Px). However, after injury, this antioxidant system often becomes overwhelmed by excessive free radicals, leading to exacerbated oxidative stress. Oxidative stress not only directly damages hepatocytes but also activates further inflammatory responses, creating a vicious cycle^4^. Hepatocyte apoptosis, a programmed cell death process, is one of the most common forms of cell death following BLI and is closely linked to both inflammation and oxidative stress. In severe cases, BLI can also cause necrosis, another form of cell death. Despite its inherent regenerative capacity, the liver initiates repair mechanisms after hepatocyte death, promoting the proliferation of residual hepatocytes through mitosis to restore the damaged area^5^. However, this regenerative process often requires several days to weeks to complete and can be inhibited if inflammation or oxidative stress is not effectively controlled. Therefore, in the early stages of injury, suppressing excessive inflammation and oxidative stress is a critical therapeutic strategy to improve the outcomes of BLI. Currently, the primary clinical management for most BLIs involves nonoperative management (NOM), focusing on monitoring, fluid replacement, and blood transfusion to maintain hemodynamic stability^6^. NOM is highly effective but lack interventions targeting pathological changes. This gap often results in prolonged recovery periods, with patients typically requiring 1 to 3 months to recover from mild to moderate BLI. Furthermore, long-term complications such as liver cirrhosis and dysfunction are common, imposing a significant burden on public health systems. These challenges are particularly pronounced in resource-limited settings, where the diagnosis and management of BLI are often more complex. Therefore, there is an urgent need for more effective interventions to complement NOM.

Hyperbaric oxygen therapy (HBOT) refers to a treatment method where the body breathes pure oxygen in an environment with a pressure higher than one atmosphere to treat various diseases. Due to its non-invasive, painless, relatively safe, and cost-effective nature, HBOT has been widely applied in clinical practice. It has shown promising therapeutic outcomes in the treatment of several trauma including traumatic brain injuries^7^, spinal cord injuries^8^, burns^9^, and fractures^10^. One of the primary mechanisms by which HBOT exerts its effects is through its anti-inflammatory and antioxidant properties. In recent years, HBOT has also been increasingly applied in the management of various liver diseases. Studies have shown that HBOT can alleviate the severity of ischemia-reperfusion injury in the liver by reducing hepatocyte necrosis and apoptosis, as well as improving sinusoidal and microvascular density indices in rat models of orthotopic liver transplantation^11^. Additionally, HBOT has been found to improve mitochondrial function following hepatic ischemia-reperfusion injury^12^. Several studies have also demonstrated that HBOT therapy can mitigate acute liver injury caused by agents such as zymosan^13^, acetaminophen^14^, and carbon tetrachloride^15^. Regarding liver regeneration, all published studies to date support the positive effects of HBOT therapy, indicating that HBOT promotes liver regeneration. These mechanisms include enhancing mitochondrial function^16^, exerting antioxidant effects, promoting hepatic angiogenesis^17^, and facilitating hepatocyte proliferation^18^. However, the effect of HBOT on BLI remains unclear.

The present study investigated the underlying hepatoprotective effect and mechanism of HBOT on BLI. The results revealed that HBOT attenuated BLI via inhibiting oxidative stress and the inflammatory response, as evidenced by serum hepatic enzymes and histological changes.

## Materials and methods

### Animals

SD rats (male, 8 weeks of age, 235-265g body weight) were provided by Laboratory Animal Management Department, Shanghai Institute of Family Planning Science. All rats were housed five animals per cage at a temperature of 21-25°C and a relative humidity of 40-60%, with 10 hours of light per day and air exchange at 20 times per hour. Food and water were freely available. A 7-day acclimatization for rats was needed before experiments began. All animals used in our study were humanely cared for according to Institutional Animal Care and Use Guidelines of the Naval Special Medical Center of PLA (No 2021070201), and the relative protocols were licensed by the Naval Special Medical Center of PLA.

### Animal model

**Figure 1:**
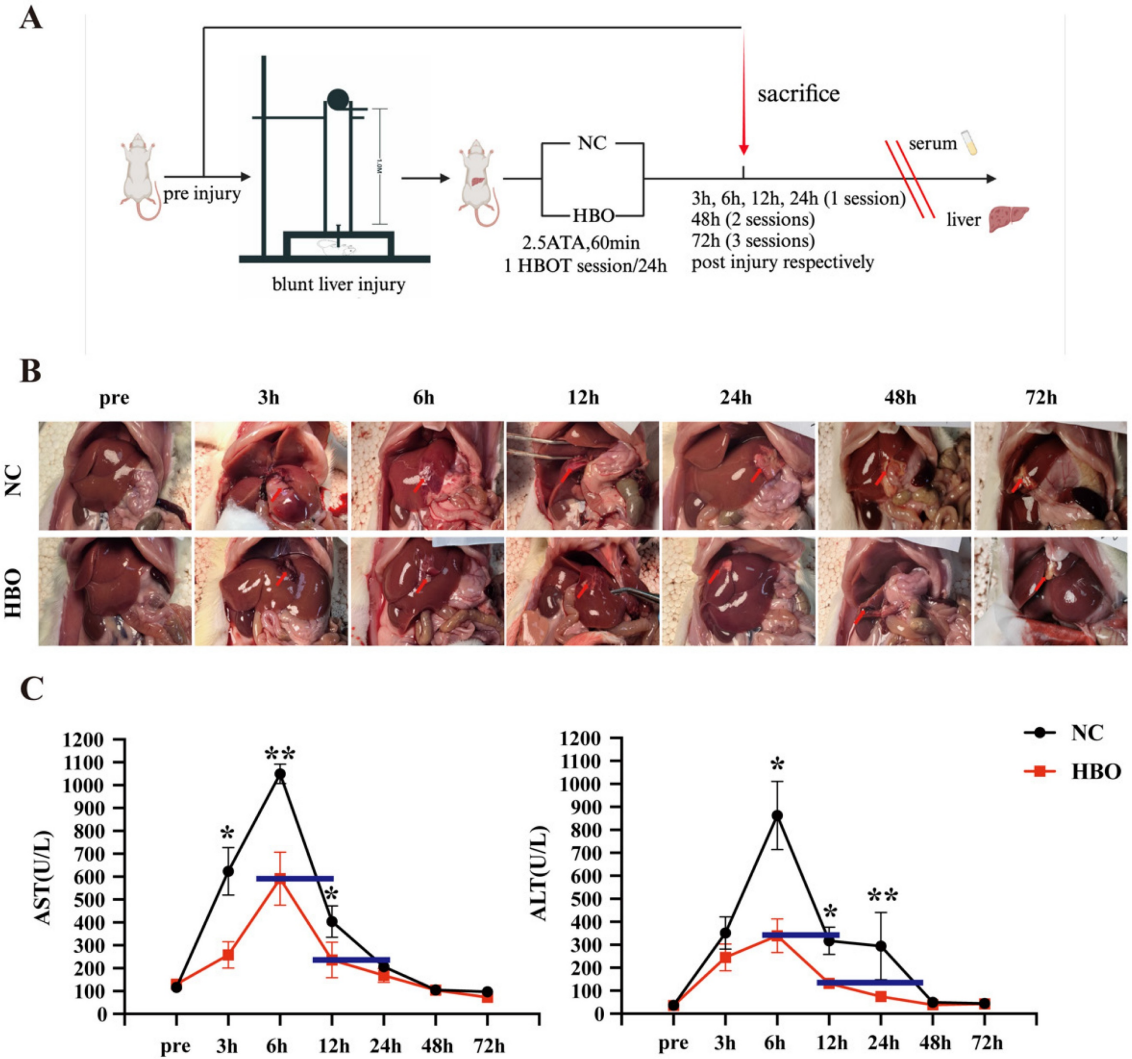
HBOT alleviated BLI in rats. (A) Experimental flowchart. (B) Gross morphology of the liver. (C) Levels of AST, ALT in plasma. Data were expressed as mean ± SEM. Independent samples t-Test was performed between two groups at each time point; ^*^p<0.05, ^**^p<0.01, ^***^p<0.001 and ^****^p<0.001. n=6.

Referring to the rat liver injury model developed by Jennifer M Cox and John E Kalns^19^, we modified and self-developed the biological impactor. The impactor consists of a steel ball, steel tube, secondary impact head, animal fixing platform, and support frame. The injury-inducing steel ball weighs 225g, with a diameter of 39mm, and the steel tube is 1m in length, while the impact head has a diameter of 1.2cm. After anesthetizing the rats with isoflurane and securing them on the animal board, the 225g steel ball is dropped freely from a height of 1m through the impactor tube, striking the impact head, which then delivers a secondary impact to the xiphoid process and the right costal margin of the rat. This procedure establishes a BLI model (Fig.1A). Kinetic parameters of the impact process are calculated as follows: the impact kinetic energy E = mgh, where g = 9.8 m/s^2, and the impact momentum P = mv. For the injury group, with the 225g steel ball and diameter of 39mm, E = 2.205 J, and P = 0.996 kg·m/s.

Liver injury severity is graded according to the American Association for the Surgery of Trauma (AAST) liver injury scale: Grade I indicates a subcapsular hematoma, with no expansion and area <10%, or a capsular tear without ongoing bleeding. Grade II involves a subcapsular hematoma, area 10-50%, or capsular tear; Grade III comprises a subcapsular hematoma >50% of the liver surface with progressive bleeding, or an intraparenchymal hematoma >10cm, or parenchymal laceration >3cm deep; Grade IV involves parenchymal disruption involving 25-75% of a hepatic lobe or 1-3 Liver segments; Grade V includes parenchymal laceration disrupting more than 75% of a lobe, or affecting 3 Liver segments within a single lobe, or major venous injury involving the retro hepatic vena cava or central hepatic veins; Grade VI indicates hepatic avulsion involving vascular injury.

### Experiment design

This is a prospective randomized controlled ecxperimental animal trial and male SD rats (235-265g) were used. After anesthetization with isoflurane (isoflurane dosage was carefully adjusted according to body weight and physiological indicators to maintain a uniform anesthesia depth, minimizing its impact on liver enzyme levels), BLI was induced using the aforementioned model. Then, rats were randomly allocated to a control group (the NC group) and an intervention group (the HBO group) to ensure the even distribution of BLI severity. The NC group received no treatment after injury, while the HBO group received immediate HBOT after injury. The rats were sacrificed after deep anesthesia with sodium pentobarbital (40 mg/kg, i.p.) at predetermined time points (pre-injury, and at 3h, 6h, 12h, 24h, 48h, and 72h post-injury). By comparing liver damage and liver function at each time point, the effects of HBOT on BLI were assessed. Hepatic Ki67 expression levels were analyzed, and TUNEL staining was performed to assess the effects of HBOT on hepatocyte proliferation and apoptosis after BLI. Levels of inflammatory markers (IL-1β, IL-6, TNF-α) and oxidative stress markers (SOD, MDA, GSH) were measured in liver tissues to evaluate the impact of HBOT on inflammation and oxidative stress in BLI.

### HBOT

Rats were administered 100% oxygen at a pressure of 2.5 atmospheres absolute pressure (ATA) in a custom-made mono-chamber (Yantai Hongyuan Oxygen Industry, Shandong, China) intended for small animals. Before compression was initiated, the monochamber was washed with 100% oxygen at a flow rate of 10L/min for 5 minutes to ensure oxygen enrichment. Compression and decompression were performed gradually within 5 minutes. Oxygen level inside the chamber following compression reached saturation of 96%~100%, as measured by an oxygen analyzer. In our study, for animals sacrificed at 3, 6, and 12 hours post-injury, a single HBOT session (2.5 ATA, 60 minutes) was administered immediately after injury. For animals sacrificed at 24, 48, and 72 hours, HBOT was administered once daily until sacrifice—resulting in 1 session for the 24-hour group, 2 sessions for the 48-hour group, and 3 sessions for the 72-hour group. Considering previous studies have shown that as for the HBOT protocol in the current study (2.5ATA for 60min, once daily), rats maintain effective ear pressure balance and tolerate HBOT well^20^, ^21^, we did not perform tympanoscopy or tympanometry in the current study. While the animals in the NC group, were placed inside the monochamber for 60 minutes without additional treatment (at 1 ATA). Temperature, measured with a temperature controller during all sessions for HBOT and control groups in the monochamber, was similar between groups.

### Histological analysis

After sacrifice, the liver tissues from the damaged areas of 3 rats were collected and fixed in 4% paraformaldehyde and then dehydrated and subsequently embedded in paraffin. Paraffin sections of approximately 5 μm thickness were prepared and needed to be dewaxed and rehydrated until staining. To reduce potential bias, all histological evaluations were performed in a blinded manner by two independent observers.

#### Hematoxylin-Eosin (HE) staining

The sections were put into a hematoxylin staining solution for 10 min and then rinsed with distilled water. The sections were added to eosin staining solution for 15 s and washed with distilled water. The sections were dehydrated by gradients of 75%, 85%, 95%, and 100% ethanol for 2 min sequence, transparent by xylene, sealed by neutral gum, then removed from the water at room temperature. The slices were sealed with neutral gum, and the water was removed at room temperature. Using optical microscopy to examine the pathological changes in liver.

#### Immunohistochemistry (IHC) and Tunel staining

IHC staining was performed on 5 μm paraffin-embedded sections of liver tissue. After dehydration, slides were incubated in Antigen Unmasking Solution Citrate Buffer pH7.4 for 20 mwa steamer for antigen retrieval and then blocked for 30 min at room temperature in 3% BSA. Then slides were incubated with primary antibodies against Ki67 (Servicebio, Wuhan, China) at 4 °C overnight. Finally, after being washed with PBS, the sections were incubated with biotinylated IgG at room temperature for 50min. Next, sections incubated with DAB (0.2 mg/ml) for 10 min in room temperature, and then mounted onto slides, dehydrated, and cover slipped. Apoptotic cells were evaluated using the terminal deoxynucleotidyl transferase (TdT) dUTP nick-end labeling (TUNEL) assay kit (Servicebio, Wuhan, China) according to a standard protocol.

### Determination of plasma transaminases

Plasma transaminase activity, including alanine aminotransferase (ALT) and aspartate aminotransferase (AST), was assessed using a full-automatic biochemical immunoassay analyzer (Roche, Switzerland) to determine the severity of liver injury.

### Determination of oxidative stress parameters

To assess the levels of GSH, SOD and MDA in the frozen liver tissues, the following steps were performed. Briefly, the frozen tissues were thawed, homogenized in lysis solution on ice using a homogenizer, and then centrifuged at 12,000 rpm for 15 min at 4 ◦C. The resultant supernatants were collected and used to detect the activities of reduced GSH, SOD and MDA using the commercially available kits (Jiancheng Bio, Nanjing, China). The assays were performed following the instructions provided by the manufacturers.

### Determination of inflammation parameters

After sacrificial, blood samples and liver tissues were collected for the detection of of IL-1β, IL-6, TNF-α concentrations using a competitive ELISA (Cusabio, Wuhan, China). All tests were performed according to the manufacturer's instructions.

### Statistical analysis

GraphPad Prism 9.0 was employed to analyze the data statistically. The results were displayed as the mean ± SEM. Unpaired two-tailed Student's t test was used for two-group comparisons at each time point. A p-value < 0.05 means statistical significance.

## Results

### HBOT alleviated BLI in rats

We successfully established an animal model of BLI (Figure 1A), with damage primarily occurring in the peripheral region of the left lobe and the middle lobe. Early injury manifestations included hemorrhage and exudation on the liver surface, subcapsular hematoma, simple parenchymal lacerations, and stellate bleeding at the site of the cracks, as well as intraperitoneal hemorrhage. According to the AAST liver injury grading system, the liver damage was classified as mild to moderate and the severity of BLI distributed evenly between the NC and HBO groups. Over time, blood clots formed, and the injured areas of the liver became darker in color. The laceration sites exhibited necrosis, accompanied by white inflammatory adhesions. As shown in Figure 1B, the anatomy of the liver in both the NC and HBO groups was normal prior to injury, with a smooth capsule, sharp edges, and a soft texture. Within 3 hours after injury, the liver was swollen, and blood-tinged fluid exuded from the surface with some blood clots, along with minor peritoneal hemorrhage. From 6 to 12 hours post-injury, some of the peritoneal blood was absorbed, and blood clots were still visible. Parenchymal lacerations began to heal. Between 24 and 72 hours, local ischemic necrosis was observed at the injury sites, with the liver becoming firmer and less elastic. Surrounding liver tissue showed signs of hyperplasia, and adhesions to the nearby gastrointestinal mucosa occurred. Compared to the NC group, no significant differences were observed in the liver between the HBO and NC groups within 12 hours post-injury. However, between 24 and 72 hours, the HBO group showed a smaller area of ischemic necrosis than the NC group.

Serum ALT and AST levels are important markers for assessing acute liver injury^22^. As was shown in Figure 1C, after BLI, both ALT and AST levels were significantly elevated compared to baseline, reaching a peak at 6 hours post-injury and then declining. By 48 hours, both groups returned to normal levels. However, at 3, 6, and 12 hours post-injury, the AST levels in the HBO group were significantly lower than those in the NC group. Specifically, AST levels in the HBO group at 6 hours were similar to those in the NC group at 10 hours, and at 12 hours, AST levels in the HBO group were comparable to those in the NC group at 24 hours. These findings suggest that HBOT not only suppressed the elevation of AST levels but also accelerated their decline following liver injury. Similarly, at 6, 12, and 24 hours post-injury, ALT levels in the HBO group were significantly lower than those in the NC group. ALT levels in the HBO group at 6 hours were comparable to those in the NC group at 12 hours, and at 12 hours, ALT levels in the HBO group were similar to those in the NC group at 40 hours. These results suggest that HBO also contributed to the lower ALT levels following liver injury. Overall, these findings demonstrated that HBOT effectively mitigated hepatocyte damage and promotes liver function recovery, particularly in the early stages following BLI.

### HBOT alleviated BLI-induced pathological changes and hepatocyte apoptosis in rats

**Figure 2:**
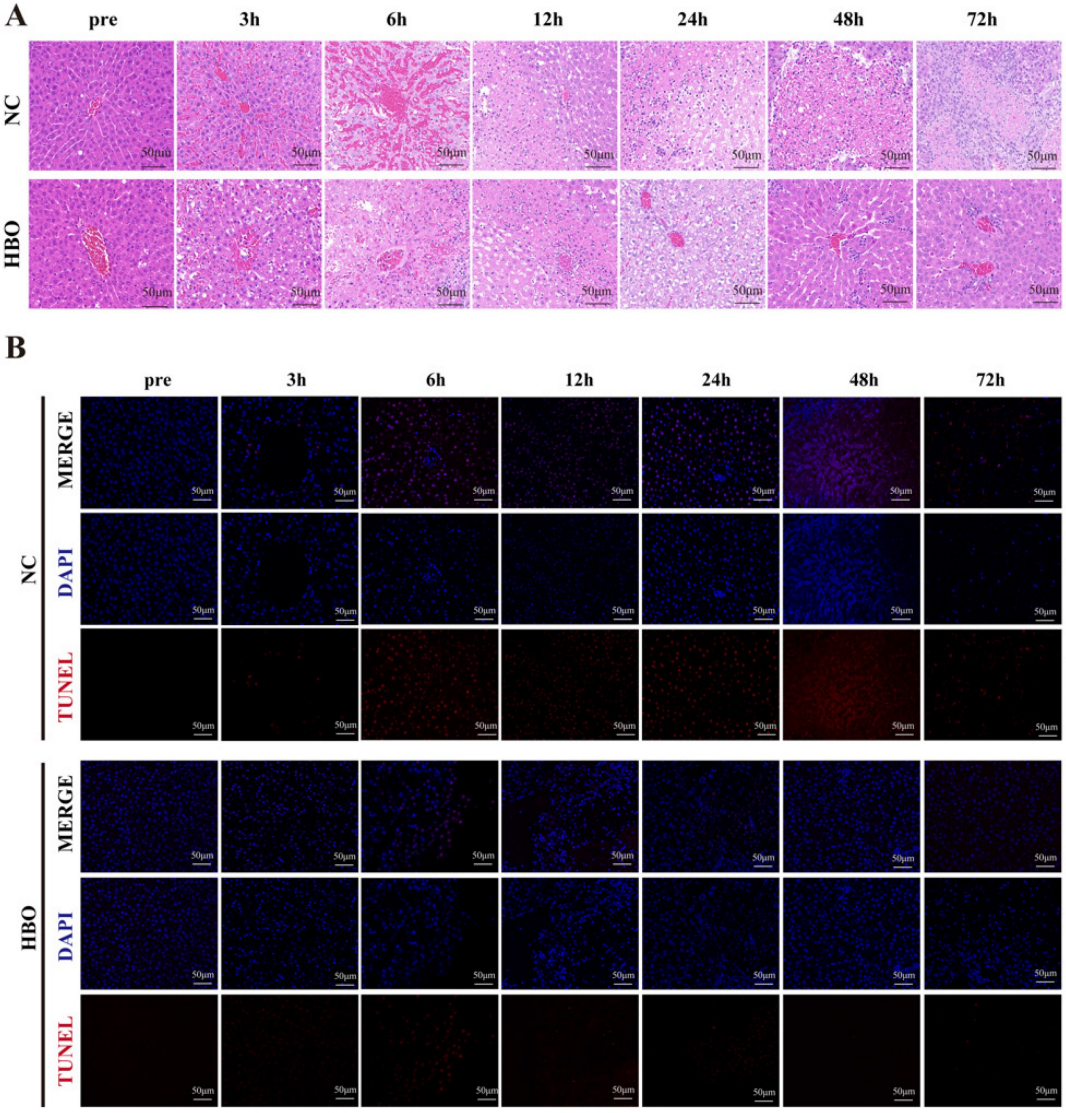
HBOT alleviated BLI-induced pathological changes and hepatocyte apoptosis in rats. (A) HE staining of the liver. (B) Tunel staining of the liver. Scale bar = 20μm. n=3.

HE staining was used to observe pathological changes at different time points. Results showed that, prior to injury, the liver tissue of rats in the NC group exhibited normal structure, with hepatocytes arranged radially around the central vein in a well-organized manner. The cell membranes were intact, cytoplasm was uniform, and the nuclei were clear. Within 6 hours post-injury, hemorrhage was the predominant feature, with destruction of the hepatic lobule structure, disordered arrangement of hepatocytes, cytoplasmic loosening, dilation of hepatic sinusoids, and accumulation of red blood cells. Between 12 and 48 hours post-injury, red blood cells in the damaged area were gradually absorbed, and hepatocyte apoptosis and even necrosis became evident. Nuclear fragmentation and dissolution of hepatocytes were observed, with extensive inflammatory cell infiltration in the necrotic areas. At 72 hours post-injury, abundant fibroblasts appeared, and the staining of fibrous matrix became more intense. Compared to the NC group, rats in the HBO group exhibited less hemorrhage within 6 hours post-injury. From 12 to 48 hours, the HBO group showed a smaller area of inflammatory cell infiltration and less hepatocyte apoptosis and necrosis compared to the NC group. At 72 hours, hepatocyte morphology in the HBOT group had largely returned to normal, with fewer inflammatory and fibroblast cells compared to the NC group (Figure 2A). These results suggest that HBOT significantly alleviates the histopathological changes associated with BLI.

To further evaluate the effect of HBOT on hepatocyte apoptosis following BLI, TUNEL staining was performed on the damaged regions of the rat liver. As was shown in Figure 2B, the results showed that in the NC group, a large number of apoptotic hepatocytes appeared in the injured region between 3 and 24 hours after BLI. In contrast, the number of apoptotic cells in the HBOT group was significantly lower than that in the NC group. Between 48 and 72 hours post-injury, the NC group exhibited a large number of necrotic hepatocytes presented with nuclear condensation, fragmentation, dissolution, and disorganized nuclear arrangement. In the HBOT group, the number of necrotic cells was also significantly lower than in the NC group. These findings indicated that HBOT can significantly suppress apoptosis and necrosis of hepatocytes in the damaged region following BLI.

### HBOT promotes cell proliferation following BLI

**Figure 3:**
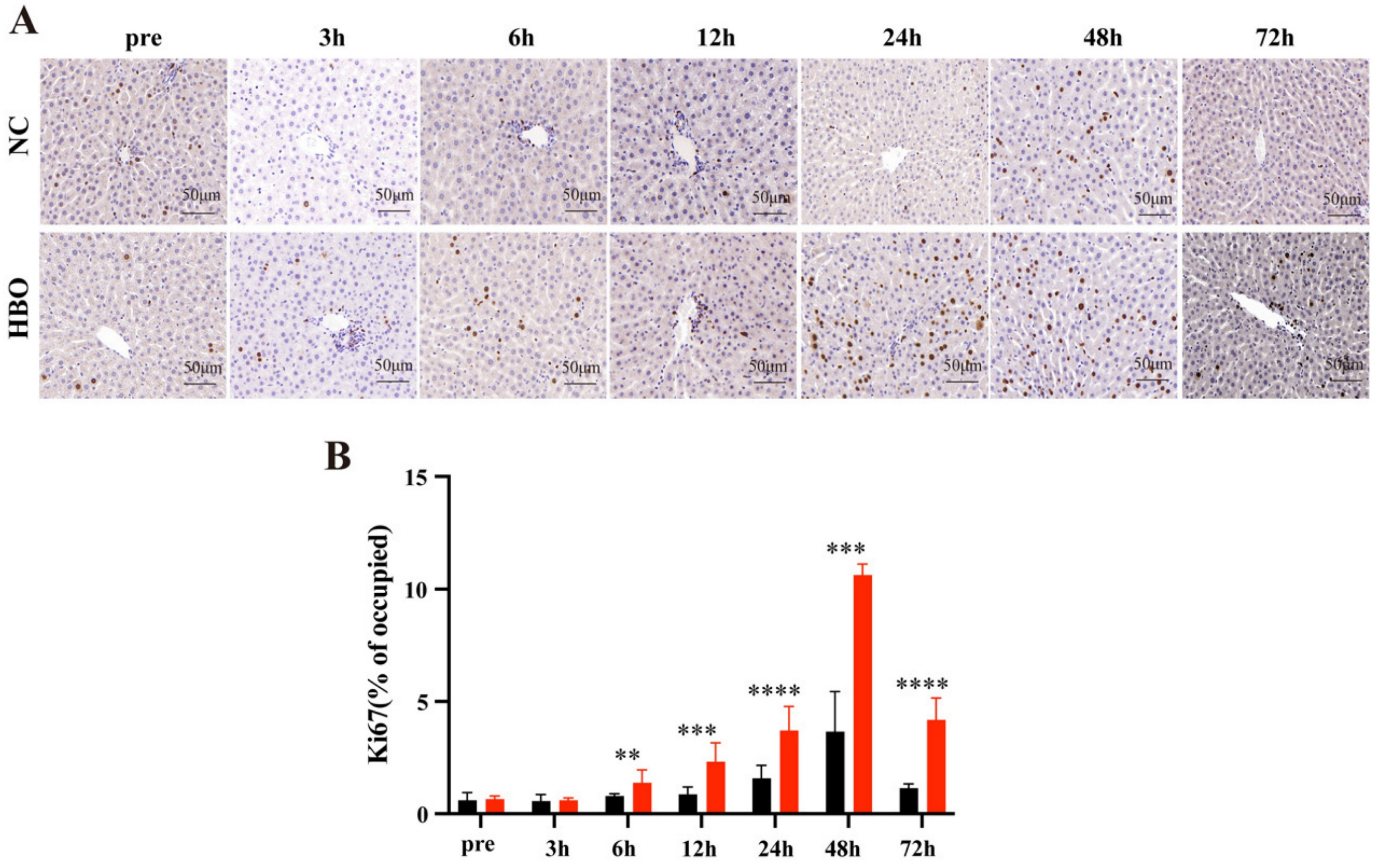
HBOT promotes cell proliferation following BLI. (A) Ki67 IHC staining of the liver. Scale bar = 20μm. (B) The Proportion of Ki67-Positive Cells. Data were expressed as mean ± SEM. Independent samples t-Test was performed between two groups at each time point; ^*^p<0.05, ^**^p<0.01, ^***^p<0.001 and ^****^p<0.001. n=3.

Ki67 is a marker of cell proliferation, and its expression in the liver is commonly used to evaluate hepatocyte proliferative capacity. IHC staining for Ki67 after liver injury revealed that the number of Ki67-positive cells began to significantly increase at 6 hours post-BLI, reaching the highest proportion at 48 hours (Figure 3A). Compared to the NC group, the HBO group exhibited a significantly higher proportion of Ki67-positive cells from 6 to 72 hours post-injury (Figure 3B), indicating that HBOT promotes cell proliferation following BLI.

### HBOT alleviated BLI-induced oxidative stress in rats

**Figure 4:**
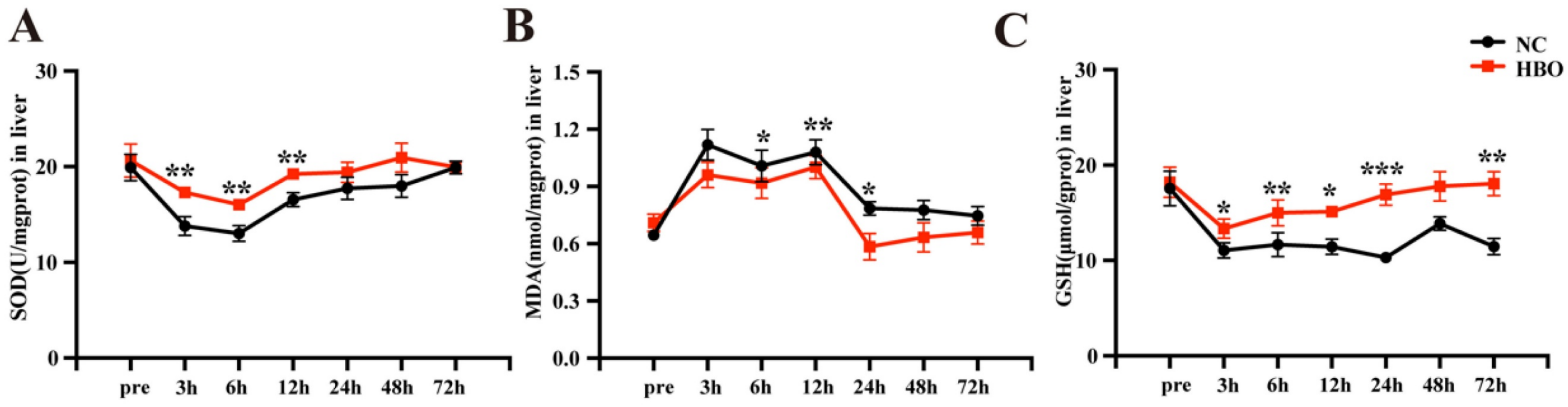
HBOT alleviated BLI-induced oxidative stress in rats. Concentration of SOD (A), MDA (B), GSH (C) in the liver. Data were expressed as mean ± SEM. Independent samples t-Test was performed between two groups at each time point; ^*^p<0.05, ^**^p<0.01, ^***^p<0.001 and ^****^p<0.001. n=6.

The antioxidant effects of HBOT have been demonstrated in several disease models. In this study, we investigated the effects of HBOT on oxidative stress in the rat BLI model. SOD is an essential antioxidant enzyme that reflects the liver's ability to counteract oxidative stress. As is shown in Figure 4A, between 3-6 hours post-injury, the SOD levels in the NC group significantly decreased, indicating a weakened antioxidant capacity and a strong oxidative stress response in the absence of intervention. In the HBO group, while SOD levels also declined, they remained significantly higher than those in the NC group, suggesting that HBOT maintained the liver's antioxidant capacity and alleviated early oxidative stress responses. Between 12-48 hours post-injury, SOD levels in the NC group showed some recovery but remained lower than those in the HBO group. In the HBO group, SOD levels nearly returned to pre-injury levels, demonstrating the significant role of HBOT intervention in enhancing antioxidant capacity and gradually mitigating oxidative stress. By 72 hours post-injury, SOD levels in both groups had returned to baseline levels. Throughout the recovery process, the SOD levels in the HBO group were consistently higher, highlighting the positive effects of HBOT. MDA is commonly used as an indicator of oxidative stress. As is shown in Figure 4B, in the NC group, MDA levels significantly increased within the first 12 hours post-injury, indicating severe oxidative stress. Although MDA levels began to decline from 24 hours onward, oxidative stress persisted and only returned to near baseline levels at 72 hours. In contrast, MDA levels in the HBO group were lower than those in the NC group at all time points, particularly at 6, 12, and 24 hours post-injury, where HBOT significantly reduced oxidative stress. Moreover, MDA levels in the HBO group returned to near-normal values earlier, by 24 hours, indicating that HBOT effectively accelerated the resolution of oxidative stress and mitigated lipid peroxidation damage. GSH, the primary intracellular antioxidant, directly scavenges free radicals. In the NC group, GSH levels significantly declined after injury, particularly at 3 and 6 hours, reflecting a marked reduction in hepatic antioxidant capacity. However, in the HBO group, HBOT intervention effectively maintained higher GSH levels. Notably, at 12 and 24 hours post-injury, GSH levels in the HBO group nearly returned to pre-injury states, suggesting that HBOT not only inhibited oxidative stress responses but also promoted a rapid recovery of hepatic antioxidant capacity (Figure 4C). Taken together, these results indicated that HBOT alleviated BLI-induced oxidative stress in rats.

### HBOT alleviated BLI-induced inflammatory response in rats

**Figure 5:**
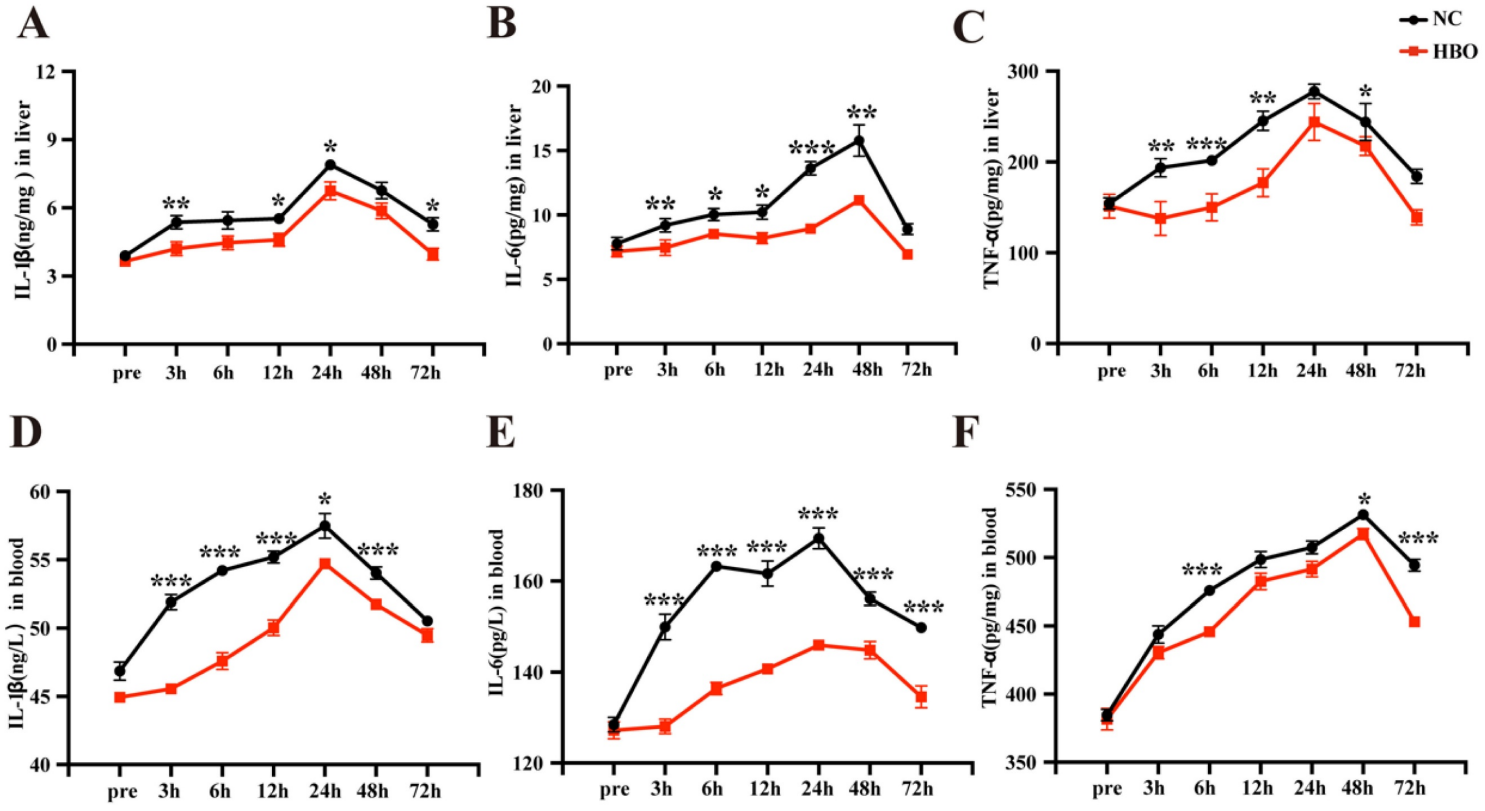
HBOT alleviated BLI-induced inflammatory response in rats. Concentration of IL-1β (A), IL-6 (B), TNF-α (C) in the liver. Concentration of IL-1β (D), IL-6 (E), TNF-α (F) in serum. Data were expressed as mean ± SEM. Independent samples t-Test was performed between two groups at each time point; ^*^p<0.05, ^**^p<0.01, ^***^p<0.001 and ^****^p<0.001. n=6.

The development of acute liver injury is closely associated with inflammatory response. The levels of IL-1β, IL-6 and TNF-α in the liver were determined using ELISA. The results showed that IL-1β levels in the NC group rapidly increased after injury, peaking at 24 hours. Although levels began to decline thereafter, they remained higher than pre-injury levels at 72 hours, indicating a prolonged inflammatory response that failed to resolve quickly. In contrast, HBOT effectively suppressed the increase in IL-1β, particularly during the early phase (3 hours), where IL-1β levels in the HBO group were significantly lower than those in the NC group. By 72 hours, IL-1β levels in the HBO group approached pre-injury values (Figure 5A). Similarly, IL-6 levels in the NC group rapidly increased following BLI, peaking at 48 hours, which indicated a significant inflammatory response. Although IL-6 levels began to decline after 48 hours, they did not fully return to pre-injury levels, suggesting a prolonged inflammatory response. HBOT significantly inhibited the elevation of IL-6, and IL-6 levels in the HBO group were markedly lower than those in the NC group before 48 hours post-injury. By 72 hours, IL-6 levels in the HBOT group approached pre-injury levels (Figure 5B). Furthermore, TNF-α levels in the NC group sharply increased after BLI, with peaks at 24 hours. HBOT significantly suppressed the rise in TNF-α levels, particularly at 3, 6, and 12 hours, where TNF-α levels in the HBO group were significantly lower than those in the NC group. By 72 hours, TNF-α levels in the HBO group were close to pre-injury levels (Figure 5C). Taken together, these results indicated that HBOT accelerated the resolution of inflammatory response in the liver. The levels of IL-1β, IL-6 and TNF-α in the serum were also determined using ELISA. Although there were slight differences, the trends in the changes of inflammatory cytokines in the serum were largely consistent with those observed in the liver (Figure 5D-5F). This further indicated that HBOT can significantly alleviated BLI-induced inflammatory response in rats.

## Discussion

Traumatic injury is one of the world's most prevalent yet neglected health concerns. BLI is among the most common types of traumatic injuries. Previous studies have demonstrated that, during the early stages following trauma, tissue damage and cell rupture release a significant amount of damage-associated molecular patterns (DAMPs)^23^, ^24^. These molecules interact with pattern recognition receptors (PRRs) on immune and other cells, activating inflammatory pathways and leading to the massive release of pro-inflammatory cytokines, thereby initiating an inflammatory response^25^. While the inflammatory response plays a critical role in physiological repair post-trauma, excessive or prolonged inflammation can result in secondary tissue damage, including apoptosis, necrosis, and exacerbated oxidative stress. Furthermore, unresolved inflammation may lead to post-traumatic fibrosis or scar formation.

Our study shows that within 3 to 24 hours post-BLI, transaminase levels increased sharply, indicating severe hepatocyte damage. HE staining results revealed extensive inflammatory cell infiltration in the injured liver tissue. ELISA results demonstrated a significant increase in the secretion of inflammatory cytokines such as IL-1β, IL-6, and TNF-α, alongside a marked decrease in SOD and GSH activity and a significant increase in MDA levels, which indicates the occurrence of oxidative stress. These findings confirm the presence of a robust inflammatory response following BLI. Previous studies also supported that in the acute liver injury, cell membrane damage could induce an inflammatory response and oxidative stress^26^, ^27^. Additionally, serum analysis indicated that BLI induced a significant peripheral inflammatory response. Among many critical organs, the liver is the largest metabolic organ in the body, and as a result, it is the central target organ for severe immune-pathophysiological damage^28^. A recent study found that after liver injury, the transcriptome of extrahepatic organs is dramatically altered, suggesting that serum metabolite-mediated cross talking networks between the liver and extrahepatic organs are very important^29^. In our study, without intervention, inflammatory responses peaked at 24 to 48 hours post-injury, and inflammatory cytokine levels remained significantly elevated above normal even at 72 hours. TUNEL staining revealed extensive apoptotic and necrotic cells post-BLI, and HE staining showed a large number of fibroblasts in the injured area at 72 hours, both of which are consequences of the inflammatory response.

Currently, the primary clinical management for most BLIs involves NOM, which includes effective approaches to prevent further disease progression yet lacks targeted strategies to modulate the inflammatory response. Although anti-inflammatory drugs such as corticosteroids^30^ and nonsteroidal anti-inflammatory drugs (NSAIDs)^31^ can reduce inflammation, they often have side effects, especially with long-term use, including liver damage, gastrointestinal discomfort, and immunosuppression, limiting their application in the management of BLI. Therefore, identifying anti-inflammatory and antioxidant interventions that do not exacerbate liver injury has attracted researchers' attention. For instance, Yilmaz N et al demonstrated that resveratrol exerts promising hepato-protective effects against blunt hepatic injury, as evidenced by decreased inflammation scores, improved hepatic histology, reduced serum transaminase activity, and enhanced modulation of regenerative and apoptotic processes^32^. Refik Ayten et al found that copper and zinc treatment reduced inflammation, lowered serum AST and ALT levels, and enhanced healing of traumatized hepatic tissue in rats with BLI^33^. Our study aims to explore more effective intervention strategies.

HBOT has shown great potential in treating traumatic injuries. In 2016, the European Committee for Hyperbaric Medicine strongly recommended HBOT as an adjunctive therapy to surgery for post-traumatic crush injuries, to be initiated as quickly as possible^34^. The co-administration of HBOT and human menstrual blood stem cell-derived exosomes synergistically promoted recovery after spinal cord injury in rats^35^. In the management of lower extremity trauma, HBOT prior to soft tissue reconstruction significantly reduced healing time and the interval from skin grafting to complete recovery^36^. The use of HBOT in traumatic brain injury is also increasingly widespread^37^. The above studies revealed that incorporating HBOT into standard trauma care may reduce injury-related complications and improve outcomes^38^. In addition, recent multicenter studies, including the HOLLT trial and pediatric TBI investigations, support the use of HBOT in trauma management, further underlining its potential clinical applicability^39^, ^40^. Furthermore, as have referred in the introduction, HBOT has demonstrated positive effects in hepatic conditions, including ischemia-reperfusion injury, post-transplant liver regeneration, and liver fibrosis. However, no study has explored the effect of HBOT on BLI yet.

Our study found that early HBOT after BLI significantly reduced the elevation of transaminase levels, shortening the time required for transaminase normalization. In the first 12 hours, the dynamic changes of inflammatory and oxidative stress responses are still evolving. Therefore, the beneficial effects of HBOT, although present (as indicated by lower biomarker levels), do not reach observable significance within this short time window. As the injury response stabilized over time, the benefits of HBOT become more evident. HBOT markedly mitigated pathological changes in the liver post-BLI, including reducing inflammatory cell infiltration, inhibiting fibroblast proliferation, suppressing hepatocyte apoptosis and necrosis, and promoting hepatocyte proliferation in the surrounding injured areas. Although the anti-inflammatory properties of HBOT have been well-documented in numerous studies^41^, ^42^, the effects of HBOT on the inflammatory response and cytokine secretion as well as the underlying mechanisms in trauma interventions remain unclear. A study showed that in a rabbit skin injury model, the expression of inflammation-related genes increased, and HBOT did not have a significant effect on gene expression^43^. However, HBOT significantly reduced inflammation mediated by traumatic brain injury^44^ and spinal cord injury^35^. In our study, we also found that HBOT significantly reduced secretion of pro-inflammatory cytokines following blunt liver injury. We speculate that the effect of HBOT on inflammatory and anti-inflammatory processes is likely depends on the type of tissue affected. In subsequent studies, we plan to conduct further investigations to thoroughly explore the underlying mechanisms by which HBOT improves the inflammatory response following BLI. While excess oxygen was once thought to induce oxidative stress, recent studies indicate that when administered within safe limits, HBOT can notably promote bone healing and repair^45^, ^46^. Consistently, our research demonstrated that HBOT significantly alleviated oxidative stress following BLI, which may be one of the primary mechanisms underlying its effects.

To the best of our knowledge, this is the first study to apply HBOT to the management of BLI. Compared with conventional treatments such as corticosteroids and NSAIDs, the HBOT has its unique advantages as a non-invasive and low-side-effect adjunct therapy. While our findings highlight the immense potential of HBOT in BLI intervention, it is important to note that the HBOT regimen discussed here is only applicable to mild to moderate BLIs that can be managed non-surgically. Hemodynamically unstable or severe BLIs still require prompt surgical intervention. HBOT may play a role in facilitating postoperative recovery in such cases, which is a potential direction for future research.

### Limitations

This study also has several limitations. Firstly, the follow-up period was limited to 72 hours, and long-term effects of HBOT on liver function and fibrosis were not assessed. Secondly, based on oxygen toxicity concerns, only a single HBOT regimen (2.5 ATA, 60 minutes, once daily) was used, and the potential benefits of alternative regimens remain to be investigated. Thirdly, mechanistic insights were limited to biomarker analyses without exploring deeper molecular pathways. Lastly, only male rats were used, which precludes analysis of potential sex-based differences in HBOT response. Future studies will extend follow-up beyond 72 hours to assess long-term liver function and fibrosis. We will test alternative HBOT regimens (varying pressure, duration, and frequency) to optimize efficacy while minimizing oxygen toxicity. Additionally, advanced molecular analyses (e.g., transcriptomics and proteomics) and the inclusion of both sexes will be employed to elucidate mechanisms and evaluate potential sex-based differences.

## Conclusions

Taken together, this study demonstrates that early HBOT following BLI significantly alleviates liver damage and promotes functional recovery. HBOT mitigates pathological liver damage, inhibits hepatocyte necrosis and apoptosis in the injured areas, and accelerates the proliferation of normal hepatocytes. These effects are achieved through the suppression of inflammatory responses and oxidative stress. Our findings offer valuable insights into the clinical management of BLI, suggesting that incorporating early HBOT into standard trauma care for patients with mild to moderate liver injuries could substantially shorten recovery times. Furthermore, this study provides additional evidence supporting the broader application of HBOT in the treatment of traumatic injuries. Of course, further studies with larger cohorts are needed to achieve a higher level of Evidence-Based Medicine and to support integration into clinical practice.
